# Risk assessment and molecular mechanism study of drug-drug interactions between rivaroxaban and tyrosine kinase inhibitors mediated by CYP2J2/3A4 and BCRP/P-gp

**DOI:** 10.3389/fphar.2022.914842

**Published:** 2022-08-22

**Authors:** Tingting Zhao, Xuening Li, Yanwei Chen, Jie Du, Xiaodong Chen, Dalong Wang, Liyan Wang, Shan Zhao, Changyuan Wang, Qiang Meng, Huijun Sun, Kexin Liu, Jingjing Wu

**Affiliations:** ^1^ Department of Clinical Pharmacology, College of Pharmacy, Dalian Medical University, Dalian, China; ^2^ Department of Pharmacy, The First Affiliated Hospital of Dalian Medical University, Dalian, China; ^3^ Dalian Institute of Chemical Physics, Chinese Academy of Sciences, Dalian, China; ^4^ Provincial Key Laboratory for Pharmacokinetics and Transport, Liaoning Dalian Medical University, Dalian, China

**Keywords:** rivaroxaban, drug–drug interaction, CYP2J2, CYP3A4, BCRP, P-gp

## Abstract

Cancer patients generally has a high risk of thrombotic diseases. However, anticoagulant therapy always aggravates bleeding risks. Rivaroxaban is one of the most widely used direct oral anticoagulants, which is used as anticoagulant treatment or prophylaxis in clinical practice. The present study aimed to systemically estimate the combination safety of rivaroxaban with tyrosine kinase inhibitors (TKIs) based on human cytochrome P450 (CYPs) and efflux transporters and to explore the drug–drug interaction (DDI) mechanisms *in vivo* and *in vitro*. *In vivo* pharmacokinetic experiments and *in vitro* enzyme incubation assays and bidirectional transport studies were conducted. Imatinib significantly increased the rivaroxaban C_max_ value by 90.43% (*p* < 0.05) and the area under the curve value by 119.96% (*p* < 0.01) by inhibiting CYP2J2- and CYP3A4-mediated metabolism and breast cancer resistance protein (BCRP)- and P-glycoprotein (P-gp)-mediated efflux transportation in the absorption phase. In contrast, the combination of sunitinib with rivaroxaban reduced the exposure *in vivo* by 62.32% (*p* < 0.05) and the C_max_ value by 72.56% (*p* < 0.05). In addition, gefitinib potently inhibited CYP2J2- and CYP3A4-mediated rivaroxaban metabolism with K_i_ values of 2.99 μΜ and 4.91 μΜ, respectively; however, it almost did not affect the pharmacokinetics of rivaroxaban *in vivo*. Taken together, clinically significant DDIs were observed in the combinations of rivaroxaban with imatinib and sunitinib. Imatinib increased the bleeding risks of rivaroxaban, while sunitinib had a risk of reducing therapy efficiency. Therefore, more attention should be paid to aviod harmful DDIs in the combinations of rivaroxaban with TKIs.

## 1 Introduction

Thrombotic complications are becoming increasingly common among cancer patients, but anticoagulant therapy always aggravates bleeding risks. The venous thromboembolism (VTE) risk of cancer patients in the tumor active period is approximately 5–6 folds greater than that of common patients (Wysokinski et al., 2019), and pulmonary embolism (PE) is the second leading cause of death in cancer patients (Hutten et al., 2000). Meanwhile, approximately 60% of cancer patients who die from PE have complications of tumor development or metastasis (Kakkar et al., 2003). In addition, the VTE recurrence ratio of patients with tumors is 2–9 folds greater than that of those without (Chee et al., 2014), and the mortality is increased by approximately three times after VTE recurrence (Khorana et al., 2007). Recent clinical guidance has suggested that no less than 6 months of anticoagulant therapy should be given for cancer-related VTE (Ca-VTE) patients; however, the consequent bleeding risk is largely increased (Farge et al., 2016). The major bleeding risk of Ca-VTE patients is increased by 2–3 times compared with that of VTE patients without tumors, which limits the clinical prognosis (Hutten et al., 2000; Chee et al., 2014). VTE shortens the overall survival of cancer patients (Kakkar et al., 2003; Song et al., 2019).

In recent years, the efficiency of direct oral anticoagulants (DOACs) applied as anticoagulant treatment or prophylaxis has been continuously confirmed, while the safety is still to be investigated. Large randomized clinical trials have also demonstrated that DOACs combined with validated risk assessment scores were a reasonable choice for the primary thromboprophylaxis of cancer patients instead of low-molecular-weight heparin (LMWH) (Song et al., 2019). Moreover, multiple clinical retrospective analyses showed that DOACs had excellent outcomes for thrombotic diseases in cancer patients; the VTE recurrence rate in cancer patients within 6 months was significantly lower than that in the LMWH group. However, the proportion of patients with major bleeding and clinically related non-major bleeding increased significantly (Li et al., 2019). The risk factors that induce bleeding in cancer patients include chronic nephrosis, thrombocytopenia, metastatic disease, and primary gastrointestinal diseases (Angelini et al., 2019). In addition to individual variation among patients, the multidrug regimen is also one of the risk factors. Rivaroxaban was recommended for the treatment of superficial vein thrombosis and VTE prophylaxis following discharge by the National Comprehensive Cancer Network of America in 2020 in the clinical practice guidelines for Ca-VTE (Streiff et al., 2020). Thus, determining the combination safety of rivaroxaban should be given high priority.

Rivaroxaban is one of the most widely used DOACs in clinical practice (Hill et al., 2020). It has been reported that rivaroxaban is the substrate of CYP2J2 and CYP3A4 and also of breast cancer resistance protein (BCRP) and P-glycoprotein (P-gp) (Mueck et al., 2014). The dominant roles of CYP2J2 and CYP3A4 in the *in vivo* metabolism (Zhao et al., 2022). However, it has been observed that simple CYP modulators, like fluconazole, did not significantly affect the pharmacokinetics of rivaroxaban in clinical trials (Mueck et al., 2013). In contrast, when CYP and transporter multitarget inhibitors were combined with rivaroxaban, like ritonavir, significant clinical changes were observed in the pharmacokinetics of rivaroxaban (Mueck et al., 2013). Thus, we speculated that transporters play a key role in the disposition of rivaroxaban *in vivo*.

Tyrosine kinase inhibitors (TKIs) compete with tyrosine protein kinases for ATP phosphorylation sites to reduce the phosphorylation of tyrosine protein kinases, which exert potent antitumor activity (Jiao et al., 2018). Imatinib, sunitinib, and gefitinib have been the mainstay treatments for various solid tumors and malignant blood diseases since they were launched in 2000 (Cheng et al., 2013; Tirumani et al., 2013; Burotto et al., 2015; Kuczynski et al., 2015; Wertheimer et al., 2015). Imatinib, which was almost the first TKI drug that gained approval by the US Food and Drug Administration (FDA), has become the first-line clinical drug for treating gastrointestinal stromal tumors and chronic myeloid leukemia (Von Mehren and Widmer, 2011; O’brien et al., 2003). However, due to the long treatment cycle, the safety of imatinib in combination with other drugs is particularly important (Nebot et al, 2010; Guilhot, 2004). As a multitargeted TKI, sunitinib exerts strong angiogenesis inhibitory activity. It was approved by the FDA in 2006 as a first-line drug for treating metastatic renal cell carcinoma, and it was also used as a second-line drug for treating imatinib-resistant patients (Kalra et al., 2015). Gefitinib is the first TKI to gain approval in the US and Japan for treating advanced non–small-cell lung cancer (NSCLC) and can significantly prolong the progression-free survival of NSCLC patients (Dhillon, 2015). It has been reported these three TKIs inhibit efflux transporters, including BCRP and P-gp, to augment anticancer activity (Dohse et al., 2010; Shen et al., 2009; Tang et al., 2012). Moreover, these three TKIs also affect CYP3A activity in reversible or irreversible modes (Filppula et al., 2012; Filppula et al., 2014). However, the combination safety of rivaroxaban with TKIs remains unknown. Therefore, the safety of rivaroxaban combined with TKIs deserves further evaluation.

The combinations of rivaroxaban with TKIs have a profound clinical foundation, and the safety may be related to pharmacokinetic targets. The present study focused on CYP2J2, CYP3A4, BCRP, and P-gp to predict the combination safety and to uncover the potential DDI mechanism based on *in vivo* and *in vitro* pharmacokinetic experiments.

## 2 Materials and methods

### 2.1 Chemicals

Rivaroxaban, sunitinib, sunitinib malate, imatinib mesylate, and NADPH were purchased from Shanghai Yuanye Bio-Technology Co., Ltd. Imatinib and gefitinib were obtained from Sigma-Aldrich (Missouri, United States). Danazol was purchased from TargetMol (United States). cDNA-expressed recombinant human CYP3A4, CYP2J2, and pooled human liver microsomes (HLM) were purchased from Cypex Ltd. (Dundee, United Kingdom). All analytical reagent-grade and high-performance liquid chromatography (HPLC)–grade solvents were from Tedia, Inc. (Ohio, United States). HPLC was performed using an Agilent MSD/MS system controller, two 1,260 series pumps, a 1,200 series autosampler, and a 1,200 series variable wavelength detector. An API 3200 triple-quadrupole mass spectrometer (Applied Biosystems, Ontario, Canada) was used for LC-MS analysis. Ionization was conducted using an electron spray interface in the positive ion mode for detecting rivaroxaban. Cell Counting Kit-8 (CCK-8) was purchased from TargetMol, United States. Dulbecco’s modified Eagle medium (DMEM) was purchased from Beijing Solarbio Science and Technology Co., Ltd. All other materials were commercially available unless otherwise stated.

### 2.2 Quantitative determination

The formation of the main metabolite of rivaroxaban in the CYP inhibition assays was quantitatively determined using HPLC. The mobile phase consisted of 60% methanol (A pump) and 40% pure water with 0.2% formic acid (B pump) with isocratic elution. The flow rate was set as 0.5 ml/min, and detection was achieved at 240 nm. Detailed methods have been described in our prior study (Zhao et al., 2022).

The LC-MS/MS method was used to quantitatively determine rivaroxaban for cell and animal experiments. LC-MS/MS analysis was performed using an API 3200 triple-quadrupole mass spectrometer (Applied Biosystems, Concord, Ontario, Canada) and an Agilent LC system Agilent HP1200 (Agilent Technology Inc. CA, United States). The column was a Hypersil ODS-BP column (150 mm × 2.1 mm, 5 μm; Dalian Elite Analytical Instruments Co. Ltd. China). The selected transition of m/z was m/z 436.1 → 145.3 for rivaroxaban (collision energy 43 eV) under the positive ion mode. The flow rate of the mobile phase was 0.4 ml/min. The mobile phase contained acetonitrile and water with 0.2% (v/v) formic acid at 65:35 (v/v) for rivaroxaban in cell experiments. The determination of rivaroxaban in the blood was achieved by gradient elution methods with a mobile phase of acetonitrile (A) and pure water with 10 mM ammonium acetate (B). The gradient program was as follows: 0–2 min, 20% A; 2–3 min, 20–80% A; 3–5.5 min, 80% A; 5.5–6.5 min, 80–20% A; 6.5–10 min, 20% A.

### 2.3 *In vitro* CYP inhibition assay

#### 2.3.1 *In vitro* CYP incubation

The inhibitory effect of the TKIs on the metabolism of rivaroxaban in recombined P450 isoforms and HLM incubations was compared by quantifiably detecting the production of the major metabolite using HPLC. The concentrations of HLM, CYP2J2, and CYP3A4 were 0.3, 0.4, and 0.6 mg mL^−1^, respectively. The selection of the rivaroxaban concentration depended on the K_m_ values of the kinetic studies (22.81, 19.37, and 46.98 μM for HLM, CYP2J2, and CYP3A4, respectively) (Zhao et al., 2022). The detailed method can be found in our previous publication (Zhao et al., 2022). Briefly, after 5-min preincubation of potential inhibitors with isoforms, NADPH was added to initiate the reaction. The reaction mixture was incubated at 37°C for 60 min, and ice acetonitrile was used for terminating the reaction. The ratio of major metabolite production of rivaroxaban in the TKI incubation group to that obtained in the control group represents the inhibitory activity.

#### 2.3.2 Initial inhibition screening

To explore the inhibitory effects of the three TKIs on rivaroxaban metabolism, three concentrations of TKIs (1, 10, and 100 μM) were used to perform the *in vitro* enzyme incubations. The ratio of the rivaroxaban main metabolite formation in the incubation with the TKIs to that without inhibitors represented the inhibitory activity.

#### 2.3.3 IC_50_ determination

The concentrations of imatinib, sunitinib, and gefitinib were 0–5 μM, 0–90 μM, and 0–10 μM, respectively, in the incubation with HLM; 0–9 μM, 0–250 μM, and 0–9 μM, respectively, in the incubation with CYP2J2; and 0–15 μM, 0–30 μM, and 0–20 μM, respectively, in the incubation with CYP3A4. The IC_50_ values were obtained by nonlinear fitting of the TKI concentration and the remaining enzyme activity.

#### 2.3.4 Reversible inhibition kinetic analysis

The incubation system with CYP3A4 included rivaroxaban (0–400 μM), potential inhibitors (imatinib: 0–10 μM; gefitinib: 0–10 μM; sunitinib: 0–20 μM), NADPH, and PBS. The incubation system with CYP2J2 included rivaroxaban (0–100 μM), potential inhibitors (imatinib, gefitinib, and sunitinib), NADPH, and PBS. The inhibition constant (K_i_) was determined using various concentrations of inhibitors and rivaroxaban. K_*i*_ was calculated by three inhibition mode formulas (competitive, noncompetitive, and mixed-mode) using Prism v.6.0 (GraphPad, San Diego, CA, United States).

#### 2.3.5 Time-dependent inhibition analysis

The two-step incubation method was performed to estimate the time-dependent inhibition (TDI). Inhibitors that caused a decrease greater than 1.5-fold in the IC_50_ value of the 30-min preincubation experiment compared with the common experiment were identified as time-dependent inhibitors.

To investigate the TDI of CYP3A4 by sunitinib, seven gradient concentrations (0–5 μM) and six time points (0–20 min) were used. It is worth noting that a higher substrate concentration than its Michaelis–Menten constant (Ki value) is required to reduce reversible inhibition. The data were then fitted to a linear regression model, which reflected the linear relation between “ln remaining activity” and “inactivation concentration” (I). The negative slope of this linear relationship reflected the observed inactivation rate (K_obs_) values, which could be plotted against I to allow the fitting of the inactivation kinetic parameters K_I_ and K_inact_ to the nonlinear least-squares regression based on Eq. 1. using Prism v.6.0 (GraphPad, San Diego, CA, United States).

$$K_{obs}=\frac{K_{inact}\times I}{K_{I}+I}$$ (1)

#### 2.3.6 Quantitative prediction of drug–drug interaction risk

Kinetic constants were included in the mechanistic static model to explore reversible inhibition and the TDI. This static model was previously developed and refined by Fahmi et al. (2008) and Isoherranen et al. (2012) to account for the inhibition of multiple P450 isoforms. In the present study, this model was designed to explore the contributions of enzyme inhibition in the prediction of DDI risk. The area under the curve ratio (AUC ratio/AUC_R_) in the presence of a pharmacokinetic DDI was used as the index, as described by Eq. 2.

$$AUC_{R}=\frac{1}{\sum_{i}^{n}\left[f_{m,P450i}\times \left(A\times B\right)\right]+\left(1-\sum_{i}^{n}f_{m,P450i}\right)}$$ (2)

Here, A is the TDI of each P450 isoform that was observed in the liver, as described by Eq. 3.

$$A=\frac{K_{\deg}}{K_{\deg}+\frac{1+K_{inact}}{I+K_{I}}}$$ (3)

$$B=\frac{1}{1+\frac{I}{K_{i}}}$$ (4)

Here, B is the reversible inhibition of each P450 isoform that was observed in the liver, as described by Eq. 4. The degradation rates (K_deg_) of CYP2J2 and CYP3A4 were 0.00026 and 0.00032 min^−1^, respectively (Cheong et al., 2017), where I represents the *in vivo* concentration of inhibitors in healthy and solid tumor patients. In addition, the fraction of rivaroxaban metabolized by CYP2J2 or CYP3A4 was input from our previous study (Zhao et al., 2022), which was 0.95 for CYP2J2 and 0.025 for CYP3A4.

### 2.4 *In vitro* transporter inhibition assay

#### 2.4.1 Cell culture

MDR1–Madin-Darby Canine Kidney cell (type Ⅱ MDCK cell), mock-MDCK cells (provided by Professor Su Zeng, College of Pharmacy, Zhejiang University, China) and ABCG2-MDCK, mock-MDCK cells (provided by Hanbio Tech (Shanghai, China)) were commonly maintained in DMEM with 10% fetal bovine serum (heat-inactivated) at 37°C with a 5% CO_2_ atmosphere. Detailed information is present in the supplementary files. The media contained a 1% non-essential amino acid solution, 100 U/ml penicillin, and 0.1 mg/ml streptomycin.

#### 2.4.2 Cell Counting Kit-8 assay for cell viability

All cells were seeded into a 96-well plate at a density of 6 × 10^4^ cells/ml. After 24 h, the complete medium was replaced with the serum-free medium containing various concentrations of TKIs for 4 h. Then, the medium containing the TKIs was discarded, and 100 μl of serum-free medium containing 10 μl of CCK-8 solution was added per well. The cells were incubated for 2 h at 37°C, and the absorbance was assessed at 450 nm using a microplate reader (Tecan, Austria). The incubation concentrations of the TKIs were determined by the clinical plasma concentrations, and the time course was determined by the subsequent experiments. The IC_50_ value was calculated using Prism GraphPad.

#### 2.4.3 Initial inhibition screening

MDR1-MDCK and ABCG2-MDCK cells were seeded in a 96-well plate at the density of 8 × 10^4^ cells/ml. After 24-h training, the complete medium was discarded and cells were washed twice with PBS buffer. Various concentrations of the TKIs were diluted using serum-free medium and given to cells for 1.5-h preincubation. Then, rhodamine123 (10 μM) or mitoxantrone (4 μM) was added into the cells with the drug-containing medium, and this was allowed to incubate for 2.5 h. Finally, the cells were washed three times with PBS buffer, and the fluorescence intensity was detected using the fluorescence reader (Tecan Trading AG, Switzerland). The excitation wavelength and emission wavelength of rhodamine 123 were 485 and 546 nm, respectively, and of mitoxantrone were 600 and 680 nm, respectively.

#### 2.4.4 Intracellular accumulation of rhodamine 123

Mock-MDCK and MDR1-MDCK cells were seeded in six-well plates and grown to 80% confluency. After approximately 24 h, the complete medium was replaced with the serum-free medium with the TKIs, and this was cultured for 1.5 h. Then, rhodamine 123 (final concentration: 10 μM) was added, and incubation was continued for 2.5 h at 37°C. At last, the medium containing rhodamine 123 was discarded, and Hochest33342 was used to stain the cell nuclei at 37°C for 10 min intracellular accumulation of rhodamine 123 was determined using an inverted fluorescence microscope.

#### 2.4.5 Bidirectional transport studies

Mock-MDCK, MDR1-MDCK, and ABCG2-MDCK cells were seeded in 24-well transwell inserts (0.33 cm^2^, 0.4 μm) at a density of 2.5 × 10^5^ cells/ml, and cells were allowed to culture for 3–5 days to form cell monolayers. The Millicell-ERS system was used to measure the TEER (Trans Epithelial Electric Resistance) values of the monolayers. The small hole with a TEER value of more than 300 Ω cm^2^ was ready for the experiments. Bidirectional transport studies were conducted using the method described previously (Jin et al., 2020). Briefly, the monolayer was washed gently using 37°C HBSS (pH 7.4) and preincubated with HBSS for 10 min at 37°C. Then, various concentrations of TKIs were added to the HBSS on either the basolateral (total volume of 800 μl) or apical (total volume of 200 μl) side of the monolayers, and the cells in the transwell inserts were allowed to further incubate at 37°C for 2 h. At last, a 100-μl solution was taken from the other side for quantitative analysis by LC-MS/MS.

### 2.5. *In vivo* pharmacokinetic experiments

To further investigate the DDI risk between rivaroxaban and the TKIs, the pharmacokinetic parameters were measured in Wistar rats (male, 160–200 g). The experiments were reviewed and approved by the Experimental Animal Centre of Dalian Medical University. All rats were obtained from Liaoning Changsheng Biotechnology Co., Ltd. All animal experiments complied with the ARRIVE guidelines and were carried out in accordance with the National Institutes of Health guide for the care and use of laboratory animals (NIH Publications No. 8023, revised 1978). All rats had free access to food and water and were fed adaptively for 7 days. The rats fasted for 24 h with free access to water before the experiments. Thirty-six rats were divided into six groups randomly. For oral administration in the pharmacokinetic experiment, the groups were as follows: group A: normal saline + rivaroxaban (2.1 mg/kg); group B: imatinib mesylate (43 mg/kg) + rivaroxaban (2.1 mg/kg); group C: gefitinib (22.5 mg/kg) + rivaroxaban (2.1 mg/kg); group D: sunitinib malate (6.0 mg/kg) + rivaroxaban (2.1 mg/kg). Blood was collected at the 15, 45, 75, 120, 180, 240, 360, 720, and 1,440 min after rivaroxaban was given. For intravenous administration in the pharmacokinetic experiment, the groups were as follows: group A: normal saline + rivaroxaban (0.4 mg/kg); group B: imatinib mesylate (10.08 mg/kg) + rivaroxaban (2.1 mg/kg). Blood was collected at the 1, 3, 5, 7, 10, 15, 30, 45, 60, 90, 120, 240, 360, and 720 min after rivaroxaban was given. After centrifugation, the plasma proteins were precipitated using acetonitrile, followed by vortexing and centrifugation.

### 2.6 Computer-aided molecular docking simulation

The CYP2J2 crystal structure homology model from the Clustal Omega web server (https://www.ebi.ac.uk/Tools/msa/clustalo/) was used to conduct docking simulations between the TKIs and rivaroxaban in SYBYL (X-1.1) (Ning et al., 2019). The PDB ID of CYP3A4, BCRP and P-gp crystal structure was 4D7D, 6VXH and 6C0V, respectively. The 3D structures of the TKIs were subjected to energy minimization using the default Tripos force field parameters, and the Gasteiger-Hückel charges were calculated for each compound. The Surflex-Dock mode was used to generate binding conformations of the TKIs with P450 isozyme and transporters. The optimal conformations were determined by their empirical functions ChemScore. The PyMOL Molecular Graphics System v.16.1.0.15350 (DeLano Scientific LLC) was used to visualize the docking results.

### 2.7 Data analysis

All pharmacokinetic parameters in the present study were analyzed using the Drug and Statistics software (DAS 2.0, Windows). In the bidirectional experiments, the apparent permeability values (Papp), efflux ratio (ER), and net flux ratio were calculated by Eqs. 5–7 (FDA, 2020). The transport of rivaroxaban was assessed to determine apparent Michaelis–Menten constants (K_m,app_). The K_m,app_ of rivaroxaban in MDCK-MDR1 and ABCG2-MDCK models was calculated by fitting a maximum effect model to the plots of the net flux ratio versus rivaroxaban concentration, according to Eq. 8 (Jacqueroux et al., 2020).

$$\text{Papp}=\left(\text{d}Q/\text{d}t\right)/\left({\text{AC}}_{0}\right)$$ (5)

$$\text{Efflux}\;\text{ratio}\left(\text{ER}\right)={\text{Papp}}_{\mathrm{AP}}/{\text{Papp}}_{\mathrm{BL}}$$ (6)

$$\text{Net}\;\text{flux}\;\text{ratio}={\text{ER}}_{\text{MDR}1-\text{MDCK}}/{\text{ER}}_{\text{mock}-\text{MDCK}}$$ (7)

$$\text{Net}\;\text{flux}\;\text{ratio}=\text{Net}\;\text{flux}\;{\text{ratio}}_{\text{max}}*{\text{C}}^{\text{h}}/\left({\text{Km,app}}^{\text{h}}+\text{Net}\;\text{flux}\;{\text{ratio}}^{\text{h}}\right)$$ (8)

where d*Q*/d*t* is the rate of drug accumulation during the study period, A is the effective growth area of cells (cm^2^), and C_0_ is the primary concentration of the drug. AP is transported from the apical to the basolateral side, and BL is transported from the basolateral to the apical side. Net flux ratio_max_ is the maximal effect, C is the concentration of rivaroxaban, and h is the Hill coefficient of the sigmoid model.

In general, the data are presented as the mean ± standard deviation and were analyzed using the Prism program (version 6.0, GraphPad, San Diego, CA). Statistically significant differences were determined using one-way ANOVA, followed by Tukey’s post hoc tests or unpaired t-tests. Statistically significant differences were indicated by *p* < 0.05.

## 3 Results

### 3.1 Effects of tyrosine kinase inhibitors on CYP-mediated rivaroxaban metabolism *in vitro*

#### 3.1.1 Initial inhibition screening

**FIGURE 1:**
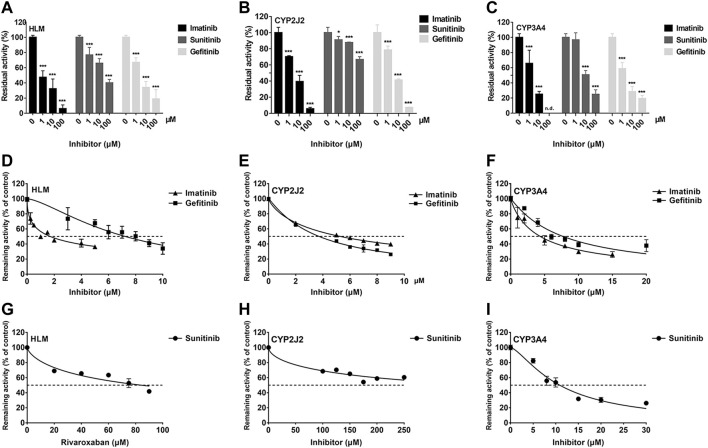
Initial inhibition screening of three tyrosine kinase inhibitors (TKIs) on CYP-mediated rivaroxaban metabolism. Inhibitory effects of three-point concentrations of TKIs on rivaroxaban metabolism with HLM **(A)**, CYP2J2 **(B)**, and CYP3A4 **(C)**. Dose–response curves of TKI inhibition with HLM **(D,G)**, CYP2J2 **(E,H)**, and CYP3A4 **(F,I)**. Results are shown as the mean ± S.D. of at least five determinations. N.D: not detectable.

To investigate the inhibition of TKIs on rivaroxaban metabolism, three concentrations, 1, 10, and 100 μM, were first used to conduct the CYP inhibition experiments. Imatinib and gefitinib showed potent inhibition of rivaroxaban metabolism in the incubation with HLM, CYP2J2, and CYP3A4 (Figures 1A, B), while sunitinib showed moderate inhibition when incubated with HLM and CYP3A4 (Figures 1A, C) and even less than 50% inhibition of CYP2J2 in a 100-μM incubation (Figure 1B). Gradient concentrations were used to determine the IC_50_ values of the three TKIs. Imatinib showed the most potent inhibition of rivaroxaban metabolism in HLM with an IC_50_ value of 1.70 μM (Figure 1D). Moreover, imatinib also exerted the strongest inhibitory effect on CYP3A4-mediated rivaroxaban metabolism with an IC_50_ value of 4.35 μM (Figure 1F). In CYP2J2-mediated rivaroxaban metabolism, gefitinib showed the strongest inhibitory activity with an IC_50_ value of 3.72 μM (Figure 1E). In general, both imatinib and gefitinib showed strong inhibitory effects on rivaroxaban metabolism mediated by CYP3A4 and CYP2J2. In contrast, sunitinib only exerted an inhibitory effect against CYP3A4, while the effect on CYP2J2 was almost imperceptible (Figures 1H, I). Detailed IC_50_ values are shown in Table 1.

**TABLE 1:** IC_50_ values of tyrosine kinase inhibitors (TKI) inhibition of rivaroxaban metabolism.

TKI	HLM	CYP2J2	CYP3A4
Imatinib	1.70 ± 0.119	5.27 ± 0.0656	4.35 ± 0.293
Gefitinib	7.236 ± 0.323	3.72 ± 0.02475	7.72 ± 0.492
Sunitinib	84.22 ± 7.343	397.70 ± 105	10.88 ± 0.450

Data are reported as μM and were obtained from five independent experiments. All data represent the mean ± S.D.

### 3.1.2 Reversible inhibition behavior analysis

**FIGURE 2:**
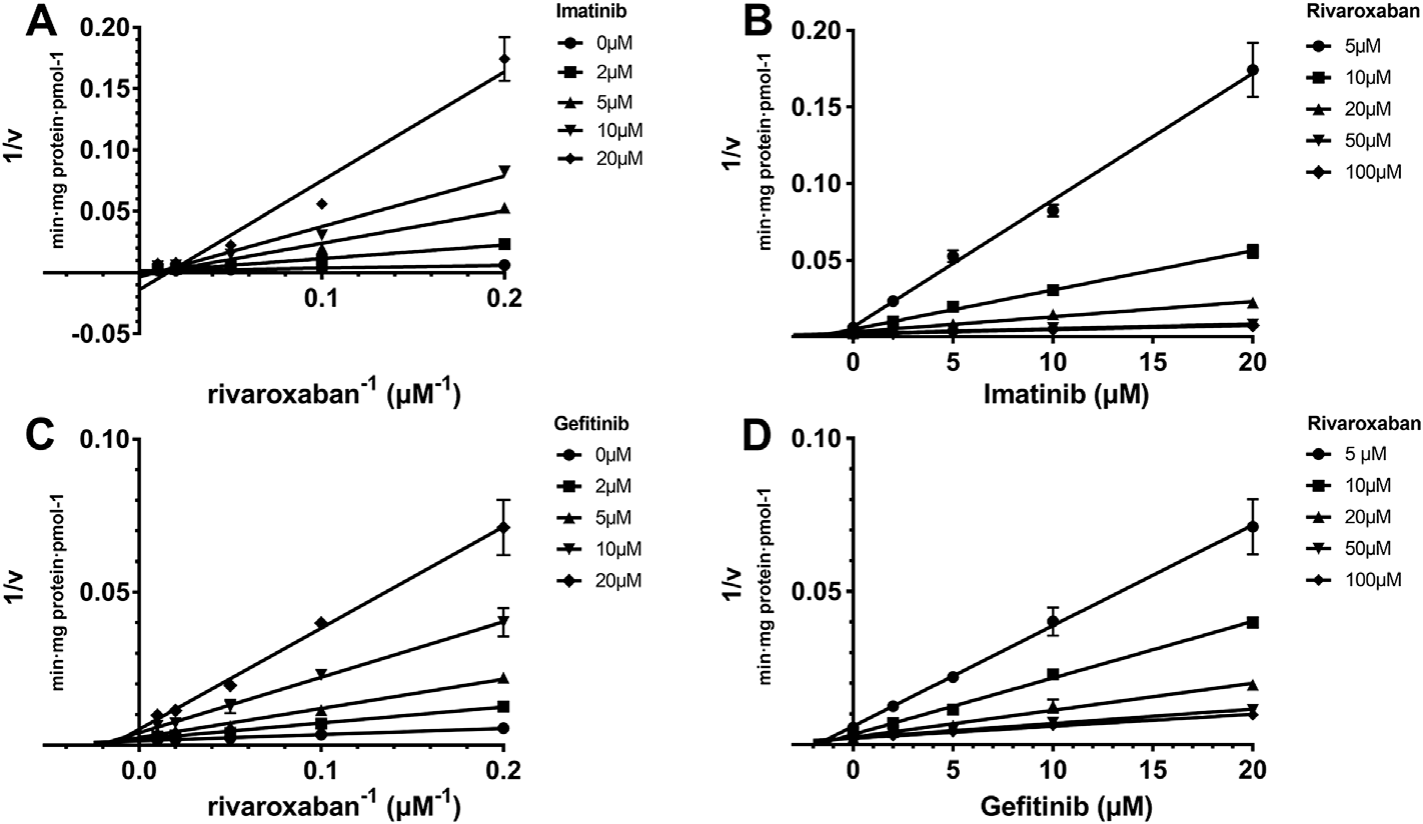
Reversible inhibition of CYP2J2 by imatinib and gefitinib. Lineweaver–Burk plots for the inhibition of imatinib **(A)** and gefitinib **(C)** on CYP2J2-mediated rivaroxaban metabolism; **(B)** and **(D)** are the corresponding Dixon plots. Data represent the mean ± S.D.

**FIGURE 3:**
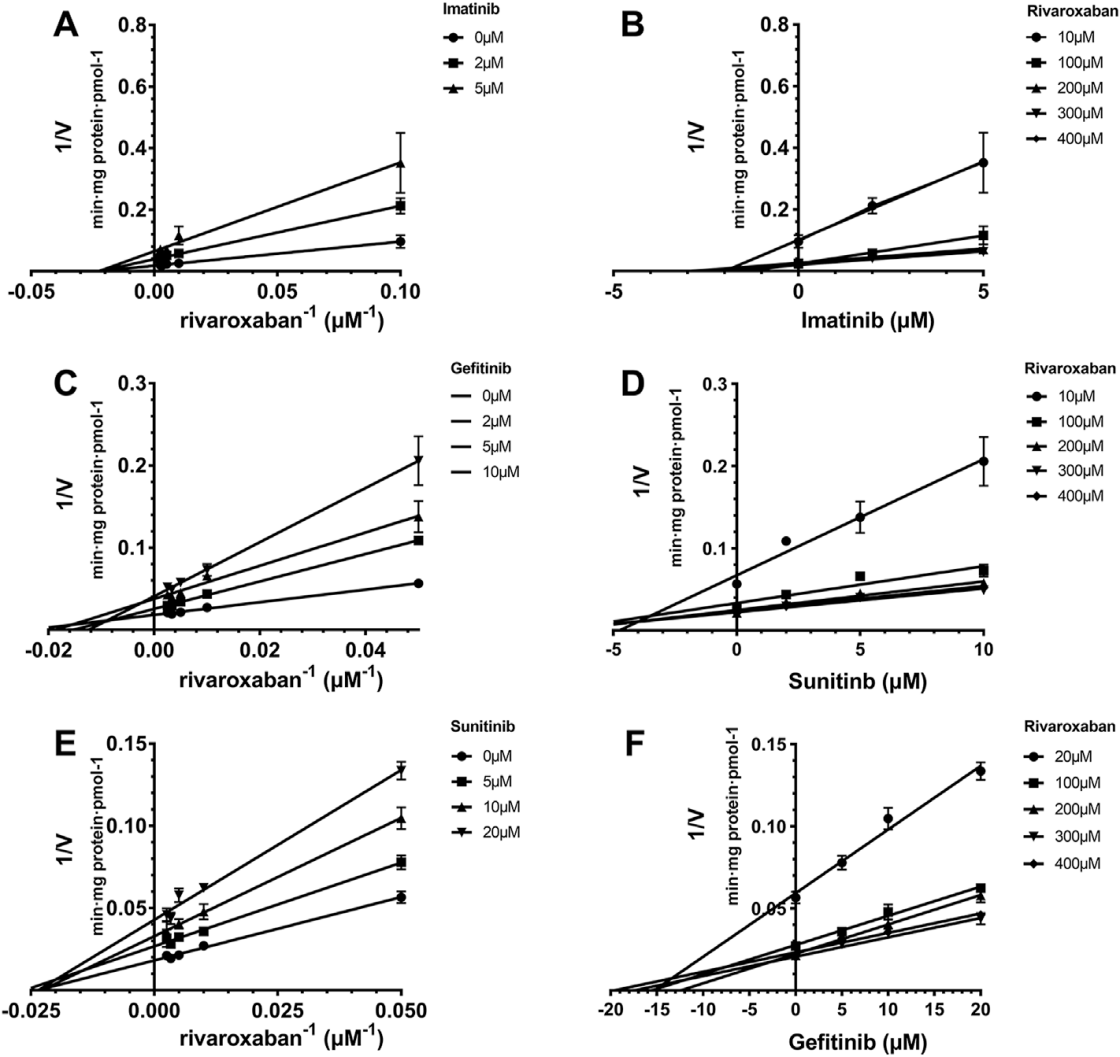
Reversible inhibition of CYP3A4 by imatinib, gefitinib, and sunitinib. Lineweaver–Burk plots for the inhibition of imatinib **(A)**, gefitinib **(C)**, and sunitinib **(E)** on CYP3A4-mediated rivaroxaban metabolism; **(B)**, **(D)**, and **(F)** are the corresponding Dixon plots. Data represent the mean ± S.D.

The K_*i*_ value of the TKIs was fitted from the kinetic curve, and the *R* ^2^ values and inhibition modes are shown in Table 2. As sunitinib did not exert more than 50% inhibition toward CYP2J2 even at 250 μM, the K_*i*_ value was not measured. All inhibitions of the three TKIs exerted on CYP2J2 and CYP3A4 were in a noncompetitive mode (Figures 2, 3). The results were corroborated by the respective Dixon and Lineweaver–Burk plots. Similar to the IC_50_ results, gefitinib and imatinib showed the strongest inhibition for CYP2J2 and CYP3A4 with K_i_ values of 2.99 and 1.92 μM, respectively.

**TABLE 2:** Reversible inhibition kinetic parameters for rivaroxaban metabolism mediated by CYP2J2 and CYP3A4.

TKIs	CYP2J2			CYP3A4		
	K_i_	Type	*R* ^2^	K_i_	Type	*R* ^2^
Imatinib	3.53 ± 0.221	Non-competitive	0.9514	1.92 ± 0.779	Non-competitive	0.9795
Gefitinib	2.99 ± 0.123	Non-competitive	0.9784	4.91 ± 0.254	Non-competitive	0.9562
Sunitinib	N.D.	N.D.	N.D.	13.24 ± 0.756	Non-competitive	0.9581

Ki was recorded as μM. Data are reported as the mean ± S.D. of three incubations. N.D: not detectable.

#### 3.1.3 Time-dependent inhibition judgment

**FIGURE 4:**
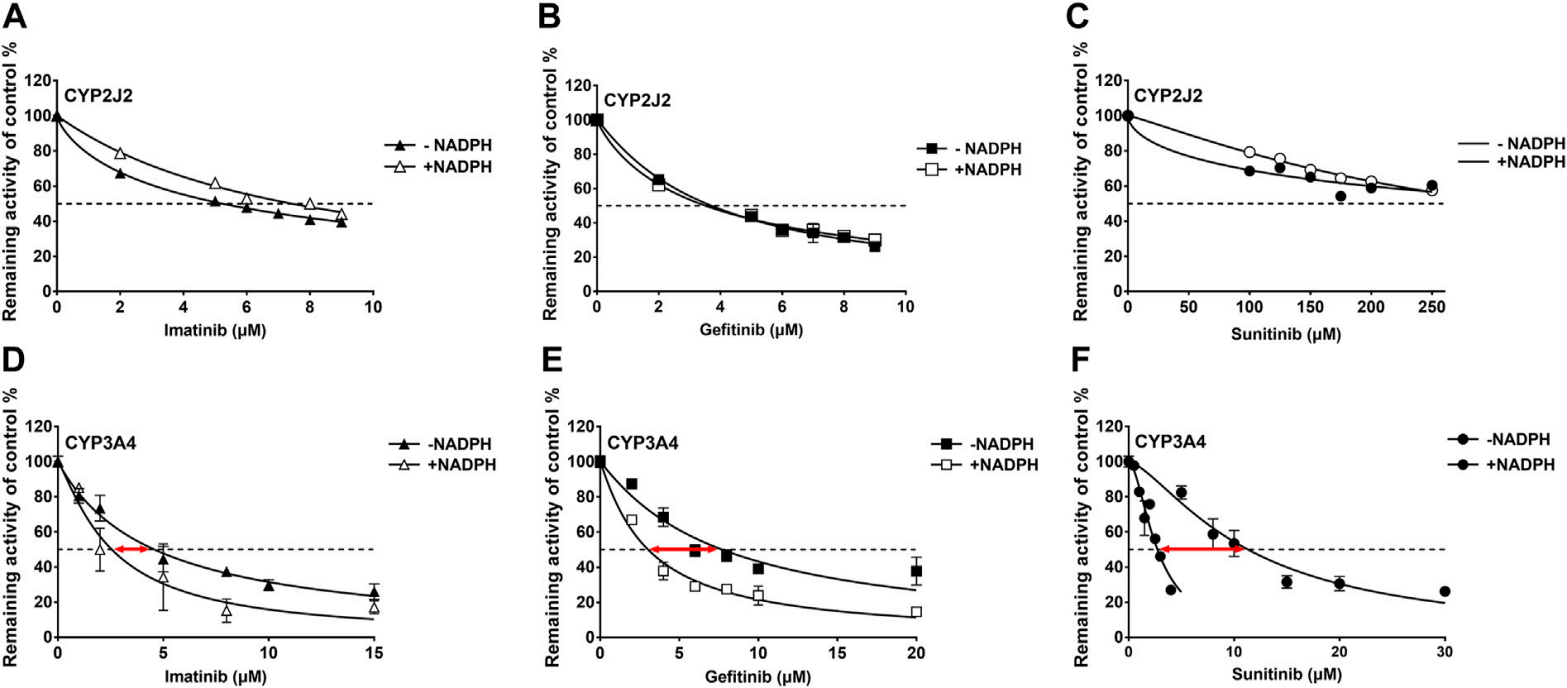
Effects of imatinib, gefitinib, and sunitinib on rivaroxaban metabolism mediated by CYP2J2 **(A–C)** and CYP3A4 **(D–F)** with or without a 30-min preincubation in the presence of NADPH. Data points are from three independent experiments.

IC_50_ shift assays of CYP2J2 and CYP3A4 were performed to explore the TDI. Compared with direct inhibition, the 30-min preincubation of TKIs with NADPH did not significantly affect the inhibition of CYP2J2, in which all IC_50_ value changes were less than 1.5 folds (Figures 4A–C; Table 3). In contrast, all IC_50_ values for the inhibition of CYP3A4 were decreased by more than 1.5 folds (Table 3). In particular, sunitinib showed the largest change in the IC_50_ shift (Figure 4F), with the IC_50_ value decreasing by 3.99 folds from 10.88 to 2.73 μM following the 30-min preincubation (Table 3).

**TABLE 3:** IC_50_ shifts initiated by preincubation of the three TKIs with NADPH in CYP2J2 and CYP3A4 incubations.

TKI	CYP2J2			CYP3A4		
	IC_50_	IC50-shift	Fold decrease	IC_50_	IC_50_-shift	Fold decrease
Imatinib	5.27 ± 0.0656	7.43 ± 0.208	0.70	4.35 ± 0.293	2.55 ± 0.370	1.9
Gefitinib	3.72 ± 0.248	3.51 ± 0.0930	1.06	7.72 ± 0.492	3.03 ± 0.218	2.55
Sunitinib	397.70 ± 105	312.90 ± 9.93	1.27	10.88 ± 0.450	2.73 ± 0.149	3.99

IC_50_ values were recorded as μM. Data are reported as the mean ± S.D. of three incubations.

#### 3.1.4 Time-dependent inhibition of CYP3A4 by sunitinib

**FIGURE 5:**
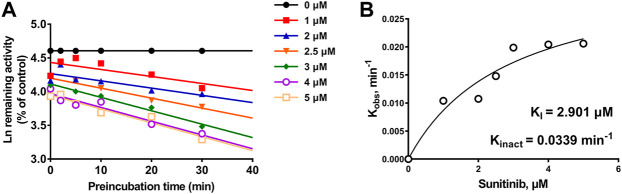
**(A)** Time- and concentration-dependent inactivation of CYP3A4-mediated rivaroxaban metabolism by sunitinib. **(B)** Observed inactivation rates (K_obs_) are plotted against the sunitinib concentration to calculate the inactivation kinetic constants K_I_ and K_inact_. Data are reported from three incubations.

Given the 3.99-fold IC_50_ decrease of sunitinib on CYP3A4 following a 30-min preincubation with NADPH, TDI constants were further determined (Figure 5A). The maximum inactivation rate (K_inact_) and the inhibitor concentration needed to produce half of K_inact_ (K_I_) were fitted using the nonlinear regression method. As shown in Figure 5B, the K_inact_ and K_I_ values of sunitinib were 0.0339 min^−1^ and 2.901 μM, respectively. The K_inact_ value indicated that approximately 3.4% of CYP3A4 was inactivated per minute when it was incubated with the saturating concentration of sunitinib.

#### 3.1.5 Estimation of the drug–drug interaction risk between rivaroxaban and TKIs based on CYP inhibition

According to the inhibition constants of the TKIs for CYP2J2- and CYP3A4-mediated rivaroxaban metabolism, the AUC changes when the TKIs were combined with rivaroxaban were predicted. Imatinib was predicted to cause a 244% increase in rivaroxaban exposure at most based on CYP inhibition (Table 4), while sunitinib and gefitinib were predicted not to cause a significant change in rivaroxaban exposure.

**TABLE 4:** Prediction of drug–drug interaction risk *in vivo* arising from inhibition of CYP2J2 and CYP3A4.

TKI	I (nM)^a^	AUC ratio^b^	AUC increase (%)
Imatinib	4,173–9,283	2.14–3.44	114–244
Gefitinib	190.2–355.8	1.06–1.11	6–11
Sunitinib	62.99–69.52	1.02	2

a I (μnM) represents the C_max_ of patients with solid tumors, which were obtained from Gschwind et al. (2005), Sparano et al. (2009), and Di Gion et al. (2011) for imatinib; Di Gion et al. (2011) for sunitinib; and Scheffler et al. (2011) for gefitinib.

b The AUC ratio was calculated based on Eqs 2–4.

### 3.2 Effects of tyrosine kinase inhibitors on transporter-mediated rivaroxaban efflux transportation *in vitro*

#### 3.2.1 Cytotoxicity of the three tyrosine kinase inhibitors on the stably transfected cells

**FIGURE 6:**
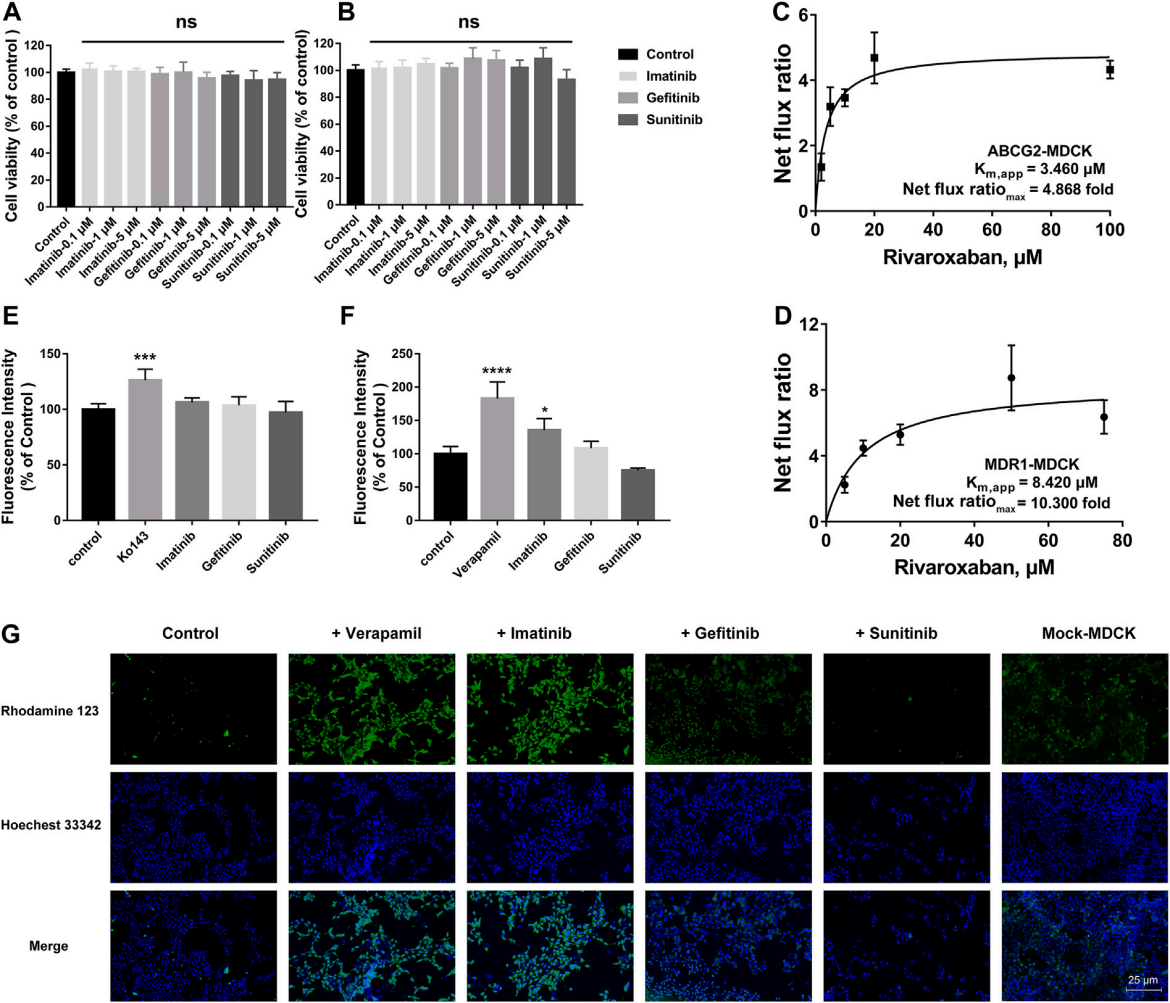
Effects of TKIs on BCRP and P-gp. Cytotoxicity of TKIs on ABCG2-MDCK cells **(A)** and MDR1-MDCK cells **(B)**. Apparent binding constant K_m_ (K_m, app_) and net flux ratio determination of rivaroxaban across ABCG2-MDCK cells **(C)** and MDR1-MDCK cells **(D)**. Inhibition screening of three TKIs on mitoxantrone efflux by BCRP **(E)** and rhodamine 123 efflux by P-gp **(F)**. Accumulation of rhodamine 123 in mock-MDCK and MDR1-MDCK cells using an inverted fluorescence microscope (100× magnification). The control group was incubated with a serum-free medium. In the inhibition screening and fluorescence imaging, 5 μM was used for the three TKIs and rhodamine 123 and 200 μM was used for verapamil. Data are expressed as the mean ± S.D. ∗*p* < 0.05, ∗∗∗∗*p* < 0.0001 compared with the control group. ns: not significant, *p* > 0.05 vs. the control group, *n* = 3.

To explore the effects of the three TKIs on the two efflux transporters, BCRP and P-gp, MDR1-MDCK and ABCG2-MDCK cells were used to conduct the subsequent experiments. The safety ranges of the three TKIs on these two stably transfected cells were determined. As shown in Figures 6A and B, all the three TKIs showed no cytotoxicity to these two cell lines in the concentration range 0.1–5 μM for 4 h.

#### 3.2.2 Efflux transportation kinetics comparison

The *in vivo* disposition of rivaroxaban was related to BCRP and P-gp; thus, the efflux transportation kinetics mediated by these two transporters were measured and compared. BCRP showed a higher affinity to rivaroxaban than P-gp, in which the K_m,app_ values were 3.460 and 8.420 μM, respectively. P-gp showed a higher ER than BCRP, in which the net flux ratio_max_ values were 10.300 and 4.868 folds, respectively (Figures 6C, D).

#### 3.2.3 Initial screening of inhibition on efflux transporters

To determine whether the three TKIs inhibited BCRP and P-gp, the fluorescence substrates mitoxantrone and rhodamine 123 were used. According to the safety range of the TKIs, 5 μM of TKIs was used. Ko 143 (20 μM) and verapamil (200 μM) were used as the positive group for inhibiting BCRP and P-gp, respectively. Ko 143 and verapamil significantly inhibited the fluorescence substrate efflux transportation mediated by BCRP and P-gp, respectively (Figures 6E, F). Imatinib potently inhibited rho-123 efflux transportation mediated by P-gp, but imatinib did not show effect on BCRP-mediated transportation. Gefitinib caused a slight increase in the intracellular rhodamine 123 fluorescence intensity in the MDR1-MDCK cells but without statistical differences. On the contrary, sunitinib decreased the intracellular rhodamine 123 fluorescence intensity but also without statistical differences.

#### 3.2.4 Effects of tyrosine kinase inhibitors on intracellular accumulation of rhodamine 123

To more intuitively observe the inhibition effect, rhodamine 123 was used as the fluorescent probe to perform fluorescence imaging in the MDR1-MDCK and mock-MDCK cells. Obvious differences were observed in the intracellular fluorescence accumulation between MDR1-MDCK and mock-MDCK cells when they were incubated with rhodamine 123. The fluorescence intensity in the MDR1-MDCK cells was significantly weaker than that in the mock-MDCK cells (Figure 6G). Verapamil, one of the classical inhibitors of P-gp, significantly increased the intracellular accumulation of rhodamine 123 in the MDR1-MDCK cells. This suggested that P-gp mediated the efflux transportation of rhodamine 123. In particular, imatinib also significantly increased the intracellular fluorescence intensity. Gefitinib also showed a slight increase in the fluorescence intensity in the MDR1-MDCK cells. On the contrary, the fluorescence intensity in cells incubated with sunitinib was similar to that in the control group.

#### 3.2.5 Effects of tyrosine kinase inhibitors on bidirectional transportation of rivaroxaban

**FIGURE 7:**
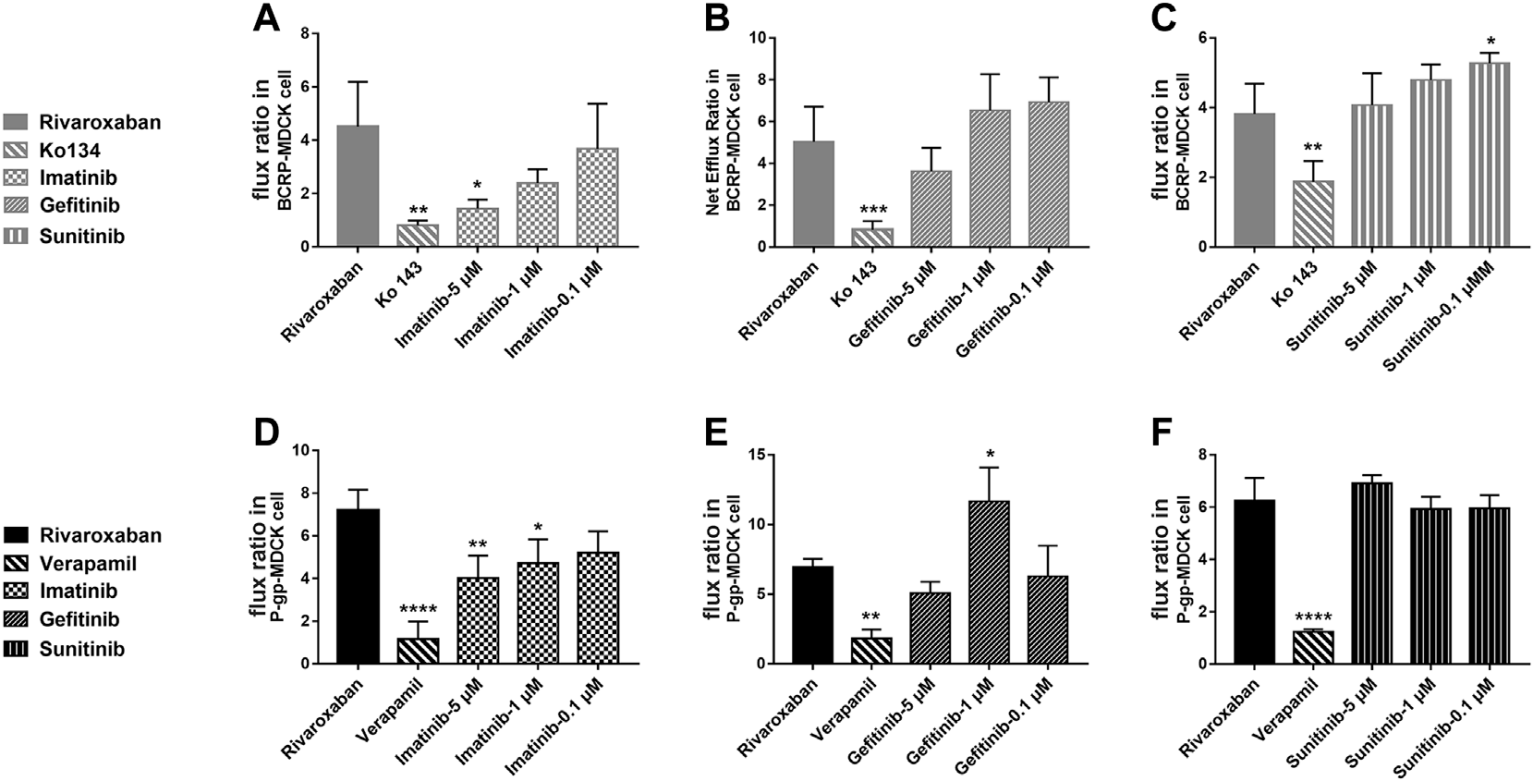
Inhibition of the three TKIs on rivaroxaban efflux transported by BCRP **(A–C)** and P-gp **(D–F)**. Data are expressed as the mean ± S.D. ∗*p* < 0.05, ∗∗*p* < 0.01, ∗∗∗∗*p* < 0.0001 compared with the control group; *n* = 3.

To study the effects of the TKIs on the efflux function of BCRP and P-gp, bidirectional transportation inhibition assays were used. Rivaroxaban was used as the substrate to conduct the bidirectional transportation study in the MDR1-MDCK, ABCG2-MDCK, and mock-MDCK cells. The net flux ratio values of rivaroxaban in the transportation mediated by BCRP and P-gp were more than 2 folds, which indicated that rivaroxaban was the substrate of these two transporters. Ko 143 and verapamil significantly inhibited rivaroxaban efflux transportation mediated by BCRP and P-gp, respectively. When rivaroxaban was incubated with 5 μM imatinib, the net flux ratio of rivaroxaban mediated by BCRP significantly decreased (Figure 7A). In addition, 1 and 5 μM imatinib significantly inhibited rivaroxaban efflux transportation mediated by P-gp (Figure 7D). Notably, the inhibition of imatinib on BCRP- and P-gp-mediated rivaroxaban efflux transportation was in a dose-dependent manner (Figures 7A, D). In contrast, 1 μM gefitinib increased the rivaroxaban efflux transportation mediated by P-gp (Figure 7E). In addition, 0.1 μM sunitinib increased the net flux ratio of rivaroxaban mediated by BCRP (Figure 7C).

### 3.3 Effects of tyrosine kinase inhibitors on rivaroxaban pharmacokinetics *in vivo*

#### 3.3.1 Effects of tyrosine kinase inhibitors on rivaroxaban pharmacokinetics by oral administration *in vivo*

**FIGURE 8:**
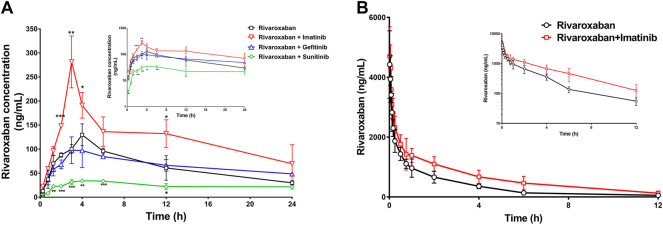
Mean plasma concentration–time curves of rivaroxaban when rivaroxaban was administered alone or coadministered with TKIs after oral administration **(A)** and intravenous administration **(B)** in rats. Data are expressed as the mean ± S.D. ∗*p* < 0.05, ∗∗*p* < 0.01, ∗∗∗*p* < 0.001 compared with the control group; *n* = 5.

To further evaluate the combination safety of rivaroxaban and the three TKIs, the *in vivo* pharmacokinetics of rivaroxaban combined with the TKIs through oral administration were measured. As shown in Figure 8A, the rivaroxaban exposure of the imatinib coadministration group was significantly increased. Coadministered with imatinib, the C_max_ value of rivaroxaban was increased from 129.7 ng/mL to 281.4 ng/mL (*p* < 0.01), which was a 119.96% increase. Imatinib oral administration also caused a 90.43% increase in the AUC value, which increased from 1.547 ug/ml h to 2.946 ug/ml h (*p* < 0.05). In addition, imatinib shortened the rivaroxaban time to peak by 1 h. On the contrary, sunitinib significantly reduced rivaroxaban exposure *in vivo* (Figure 8A). The AUC value of the group co-administrated with sunitinib coadministration group was approximately 37.68% (*p* < 0.01) to that of the rivaroxaban alone group. The C_max_ value of rivaroxaban was deceased by 72.56% (*p* < 0.01). The gefitinib coadministration group did not show obvious changes in rivaroxaban exposure. Gefitinib shortened the time to peak by 1 h. Detailed pharmacokinetic parameters are shown in Table 5.

**TABLE 5:** Pharmacokinetic parameters of rivaroxaban per p.o. administration.

Parameter	Rivaroxaban	Rivaroxaban + imatinib	Rivaroxaban + gefitinib	Rivaroxaban + sunitinib
AUC(0-t)	1.547 ± 0.268	2.946 ± 0.647^*^	1.587 ± 0.145	0.583 ± 0.101^**^
C_max_	129.7 ± 22.70	281.4 ± 54.05^**^	106.8 ± 23.60	35.60 ± 2.426^**^
T_max_	4	3	3	4

AUC(0-t) was recorded as ug/mL·h, C_max_ was recorded as ng/mL, and T_max_ was recorded as h. Data are expressed as the mean ± S.D.

* *p* < 0.05.

** *p* < 0.01 compared with control; *n* = 5.

#### 3.3.2 Effects of imatinib on rivaroxaban pharmacokinetics by intravenous administration *in vivo*

To further investigate the DDI mechanism between imatinib and rivaroxaban, the *in vivo* pharmacokinetics of rivaroxaban combined with imatinib through intravenous administration were measured. The mean plasma concentrations of rivaroxaban were weakly increased by imatinib but without statistical differences (Figure 8B). In addition, the AUC value was increased by 72.99% (4.648 ug/ml h to 8.041 ug/ml h; *p* < 0.01). Moreover, the CL value of rivaroxaban was decreased from 0.083 L/h/kg to 0.047 L/h/kg (*p* < 0.001), which was reduced by 43.38%. Furthermore, the t_1/2_ and C_max_ values were slightly increased but without statistical differences (*p* > 0.05). Detailed pharmacokinetic parameters are shown in Table 6.

**TABLE 6:** Pharmacokinetic parameters of rivaroxaban per i.v. administration.

Parameter	Rivaroxaban	Rivaroxaban + imatinib
AUC(0-t)	4.648 ± 0.446	8.041 ± 1.510^**^
t_1/2_	2.444 ± 0.757	3.636 ± 2.274
CL	0.083 ± 0.007	0.047 ± 0.014^***^
C_max_	3.937 ± 0.780	4.301 ± 0.780

AUC(0-t) was recorded as ug/mL·h, t_1/2_ was recorded as h, CL was recorded as L/h/kg, and C_max_ was recorded as ug/mL. Data are expressed as the mean ± S.D.

* *p* < 0.05.

** *p* < 0.01.

*** *p* < 0.001 compared with control; *n* = 5.

### 3.4 Molecular docking simulations

Molecular docking simulations were used to elucidate the binding conformations for the interactions between the TKIs and CYP2J2, CYP3A4, BCRP, and P-gp. In the docking simulations between CYP2J2 and the TKIs, gefitinib had the lowest ChemScore value, followed by imatinib and then sunitinib. The ChemScore ranking was consistent with the inhibition intensity. Likewise, imatinib had the lowest ChemScore value in the docking simulation with CYP3A4, followed by gefitinib and then sunitinib, which was also consistent with the inhibition intensity of these TKIs on CYP3A4. Moreover, imatinib had the lowest ChemScore values in the docking simulations with BCRP and P-gp, which was in accordance with the most potent inhibition among these three TKIs on BCRP and P-gp. Detailed ChemScore values are shown in Table 7. Molecule docking simulations of the TKIs with CYPs are shown in Supplementary Figure S1.

**TABLE 7:** Comparison of ChemScore values of TKIs binding to CYP2J2, CYP3A4, BCRP, and P-gp.

TKIs	CYP2J2	CYP3A4	BCRP	P-gp
Imatinib	−30.296	−49.611	−31.524	−29.995
Gefitinib	−33.232	−38.264	−30.913	−29.384
Sunitinib	−25.039	−38.026	−28.127	−25.905

## 4 Discussion

The medication safety of rivaroxaban has mostly focused on patients coadministered with cardiovascular drugs, while little attention has been given to cancer patients. Abnormal hemodynamics and physiological disorders of cancer patients lead to a high incidence of thrombotic diseases. Thus, anticoagulation prevention or treatment is necessary, and the combination of rivaroxaban with anticancer drugs has a profound clinical basis. The present study found that clinically significant DDIs exist in the combination of rivaroxaban with imatinib and sunitinib. Imatinib significantly increased rivaroxaban exposure *in vivo* and also caused a change in the pharmacokinetic absorption parameters, T_max_ and C_max_, which may increase bleeding risks (Figure 8). Imatinib showed more than 50% inhibition on BCRP-mediated rivaroxaban efflux at a concentration of 5 μM and also inhibited approximately 20% P-gp-mediated rivaroxaban efflux in a dose-dependent manner (Figures 7A, D). The intestinal concentration of imatinib was predicted to be in the range of 3.2–6.4 mM according to the FDA guidance document published in 2012, under which imatinib may interact with rivaroxaban based on BCRP and P-gp in the intestine. In addition, the inhibition constants of imatinib on CYPs (K_i_ and IC_50_) were lower than its plasma concentration (Tables 1, 2, and 4), which may inhibit rivaroxaban metabolism *in vivo* (Table 6). In contrast to imatinib, sunitinib significantly decreased rivaroxaban exposure when they were combined, which may be caused by sunitinib promoting BCRP efflux transportation. The promotion of sunitinib at 0.1 μM was stronger than that at 1 and 5 μM (Figure 7C). The promotion at 0.1 μM was stronger than that at 1 and 5 μM (Figure 7C). According to the sunitinib clinical oral dose of 50 mg/d, the maximum plasma concentration was approximately 70 nM (Di Gion et al., 2011), which was similar to the concentration of promoting BCRP but much higher than that of inhibiting CYP2J2 or CYP3A4 (Tables 1, 2 and Tables 3, 4). Therefore, we speculated that sunitinib decreased exposure by promoting BCRP efflux transportation and then increasing rivaroxaban excretion. Lafaie et al. also evaluated the DDI risks in combinations of DOACs with TKIs using *in vitro* cell models (Lafaie et al., 2022). Imatinib was also predicted to have intestinal DDI risks based on P-gp when combined with rivaroxaban. While sunitinib showed little inhibition on P-gp, the intestinal DDIs of sunitinib with rivaroxaban might be less risky. The prediction was in accordance with our results. In particular, endogenous canine transporters of MDCK, such as canine Mdr1, may influence the *in vitro* results. Therefore, all results obtained in overexpression cells should be compared with the results of the mock-MDCK group to eliminate the effect of carrier cells.

Regarding the combination safety of rivaroxaban, numerous studies have focused on the metabolic enzyme and transporter, but the contribution of the target itself remains unknown. Cheong et al. (2017) predicted a moderate DDI risk in the combinations of rivaroxaban with antiarrhythmic agents, amiodarone and dronedarone, based on the mechanism of inhibition on CYP2J2, CYP3A4, and P-gp *in vitro*. In addition, the antiplatelet drug ticagrelor was reported to increase the AUC of rivaroxaban by two folds in rats (Chong et al., 2020). Another DDI study showed that enalapril increased the C_max_ and AUC values of rivaroxaban by 20%, which suggested to decrease the rivaroxaban dose when it was combined with enalapril for the treatment of hypertensive patients with atrial fibrillation (Zheng et al., 2019). However, there were many pharmacokinetic targets in rivaroxaban-related DDIs, and their contributions remained unclear. It has been reported that ketoconazole or ritonavir, potent CYP3A4 and P-gp dual inhibitors, cause clinically significant and harmful DDIs with rivaroxaban (Mueck et al., 2013). Notably, ketoconazole and ritonavir could also potently inhibit CYP2J2 and BCRP (Lee et al., 2012; Kaspera et al., 2014; Vermeer et al., 2016). Combined with our results, in which CYP2J2 showed approximately 39-fold catalytic efficiency to CYP3A4 and BCRP showed a higher affinity than P-gp, the roles of CYP2J2 and BCRP among the DDIs related to rivaroxaban cannot be excluded (Zhao et al., 2022). In addition, approximately 14% of the dose is eliminated via hydrolysis of the amide bonds (Mueck et al., 2013). Thus, human carboxylesterase may also contribute to the metabolism of rivaroxaban, which is worthy of further study.

Although there are numerous DDI studies related to rivaroxaban, it is still difficult to gauge the relative contribution of the different mechanisms to DDIs. Since various pharmacokinetic targets participate in the disposition of rivaroxaban, we have evaluated every single factor quantitatively *in vitro* and overall *in vivo*. Our results showed that clinically relevant DDIs may occur in the combination of rivaroxaban with imatinib and sunitinib. Our results may be of great value to guide the risk assessment of rivaroxaban combined with TKIs. Thus, clinical studies are warranted to investigate these harmful interactions. However, at present, the relative contributions of the different mechanisms to the DDI risk cannot be evaluated. *In vitro* cell models or CYP incubation assays cannot simulate the specific organs or physiological processes. Therefore, it is of potential significance to develop the physiologically based pharmacokinetic model (PBPK) of rivaroxaban based on specific biological factors. The metabolic activity of the enzyme and the transport activity of the transporter could be detected using probe drugs to develop individualized combined medication DDI risk prediction models, which would predict the contributions of various mechanisms in DDIs. Moreover, the data obtained from human clinical trials based on the present *in vitro* and *in vivo* results will be of great significance for PBPK to predict DDI risks between rivaroxaban and TKIs, which is the focus of our further study. Those results would contribute to the realization of formulating individualized dosing regimens. In fact, Cheong et al. have developed and verified such a model, which would make the basic study more clinically significant (Cheong et al., 2019).

In conclusion, the combination safety of rivaroxaban with TKI drugs was comprehensively evaluated *in vivo* and *in vitro*. Imatinib significantly increased rivaroxaban exposure by inhibiting CYP2J2, CYP3A4, BCRP, and P-gp, while sunitinib significantly decreased rivaroxaban exposure by promoting the efflux transportation of rivaroxaban mediated by BCRP. Therefore, clinical studies are warranted to investigate these harmful interactions.

## Data Availability

The raw data supporting the conclusions of this article will be made available by the authors, without undue reservation.
