# Supplementary material for: Risk assessment and molecular mechanism study of drug-drug interactions between rivaroxaban and tyrosine kinase inhibitors mediated by CYP2J2/3A4 and BCRP/P-gp
Source: Front Pharmacol. 2022 Aug 22;13:914842. doi: 10.3389/fphar.2022.914842 (PMC9441481; doi:10.3389/fphar.2022.914842)
Supplement: Supplementary file 1 [file DataSheet1.pdf]

Risk Assessment and Molecular Mechanism Study of Drug-drug Interactions Between Rivaroxaban and Tyrosine Kinase Inhibitors Mediated by CYP2J2/3A4 and BCRP/P-gp

Tingting Zhao[a #], Xuening Li[a #], Yanwei Chen[c, #], Jie Du[a], Xiaodong Chen[a], Dalong Wang[a], Liyan Wang[c], Shan Zhao[d], Changyuan Wang[a, b], Qiang Meng[a, b], Huijun Sun[a, b], Kexin Liu[a, b], Jingjing Wu[a, b*]

[a]Department of Clinical Pharmacology, College of Pharmacy, Dalian Medical University, Dalian 116044, China.
[b]Provincial Key Laboratory for Pharmacokinetics and Transport, Liaoning Dalian Medical University, Dalian, Liaoning, China.
[c]Department of Pharmacy, the First Affiliated Hospital of Dalian Medical University, Dalian 116011, China.
[d]Dalian Institute of Chemical Physics, Chinese Academy of Sciences, Dalian 116023, China.
[#] These authors contributed equally to this work.

*** Correspondence:**
Dr. Jingjing Wu
ORCID: 0000-0002-5046-9996
Department of Clinical Pharmacology, College of pharmacy
Dalian Medical University
9 West Section, Lvshun South Road, Lvshunkou District, Dalian 116044, China.

The establishment methods of transfected cells.

The establishment of ABCG2-MDCK cells: The lentivirus containing HBLV-NC-PURO (control) and MDCK-h-ABCG2-HIS-PURO (target) was used to transfect MDCK cells. After 48 h of infection, uninfected cells were killed by adding and maintaining 3 μg/ mL puromycin. Finally, the stable strain of ABCG2 with stable expression was obtained under the maintenance of puromycin. The overexpression effect was detected in MDCK cells by qPCR, and the overexpression fold was about 119484 times.

The establishment of MDR1-MDCK cells: The plasmid pcDNA3.1(+)/MDR1 was transfected into MDCK cell line using LipofectamineTM 2000 transfection reagent. Several stable transfected clones were obtained after selection with G418. The mRNA and protein levels were detected by using RT-PCR and Western blot, respectively(LiuYu and Zeng, 2009).

Supplementary Figure

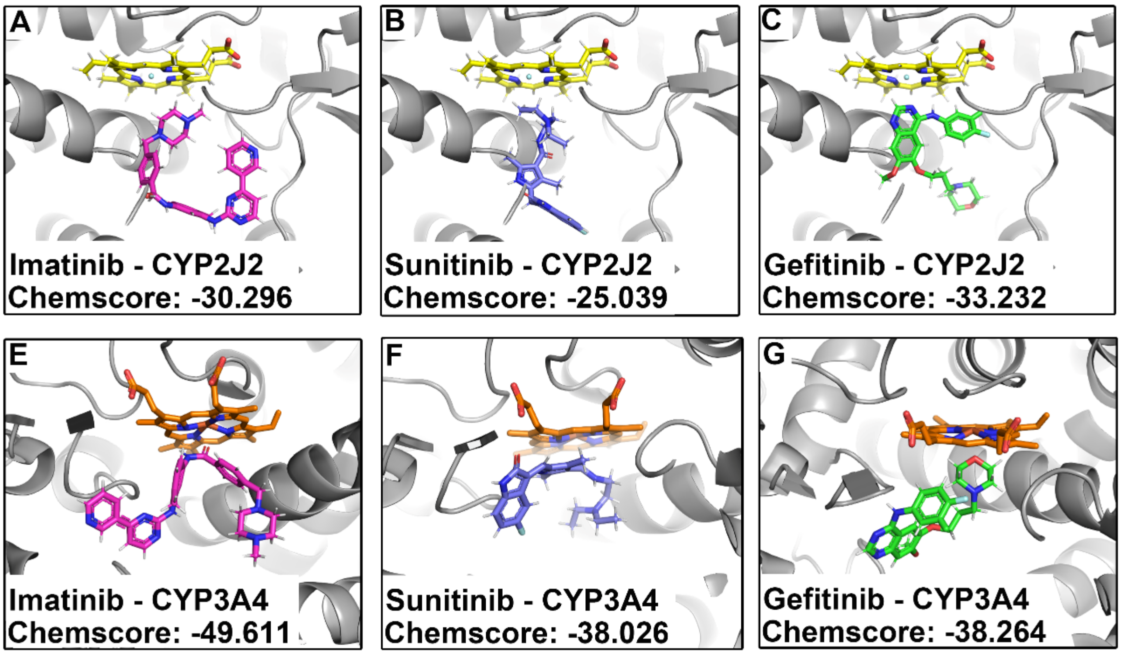

Figure S1. Molecule docking simulations of imatinib(A), sunitinib(B) and gefitinib(C) with CYP2J2, and (D, E and F) with CYP3A4, respectively. Thanks again for your suggestion.

Liu, Y., Yu, C. N. and Zeng, S. (2009). Establishment of Madin-Darby canine kidney cell line with high P-glycoprotein expression. *Journal of Chinese Pharmaceutical Sciences*, 44(21), 1608-1613.
